# Electrophile-Dependent Reactivity of Lithiated *N*-Benzylpyrene-1-Carboxamide

**DOI:** 10.3390/molecules27123930

**Published:** 2022-06-19

**Authors:** Magdalena Ciechańska, Anna Wrona-Piotrowicz, Karolina Koprowska, Anna Makal, Janusz Zakrzewski

**Affiliations:** 1 Department of Organic Chemistry, Faculty of Chemistry, University of Lodz, Tamka 12, 91-403 Lodz, Poland; magdalena.ciechanska@chemia.uni.lodz.pl (M.C.); karolina.koprowska@edu.uni.lodz.pl (K.K.); janusz.zakrzewski@chemia.uni.lodz.pl (J.Z.); 2 Biological and Chemical Research Center, Faculty of Chemistry, University of Warsaw, Żwirki i Wigury 101, 02-089 Warsaw, Poland; am.makal@uw.edu.pl

**Keywords:** pyrene carboxamide, azaolympicene, lithiation, carbolithiation, fluorescence

## Abstract

In this paper, we describe the lithiation of *N*-benzylpyrene-1-carboxamide with RLi-TMEDA. We found that the reaction outcome strongly depends on the electrophile used in the quenching step. The electrophile can be introduced at either the benzylic position or at the C-2 position in the pyrene nucleus. Furthermore, when H^+^ was used as the quencher, the product of the intramolecular carbolithiation of the pyrene K-region was formed. Dehydrogenation of the obtained compound with DDQ allowed the synthesis of a novel nitrogen polycyclic compound with an aza-benzo[c,d]pyrene (azaolympicene) skeleton. Attempts to extend the reaction scope to the amides substituted in the phenyl ring **8a** and **8b** gave an unexpected result. The reaction of both compounds with BuLi gave 1-valerylpyrene (**9**) in good yield. Photophysical properties, including absorption spectra, emission spectra and quantum yields of the emission of selected products, were studied and discussed.

## 1. Introduction

Aromatic amides are versatile starting materials for the syntheses of various organic molecules. The amido group activates and directs different transition metal-catalyzed C-H activation [1,2,3,4] and lithiation reactions [5,6,7,8,9,10]. Furthermore, this group can be easily transformed into a variety of other functional groups [11,12,13,14,15,16,17,18,19,20,21]. 

Pyrene-1-carboxamides are readily available compounds [22], which exhibit interesting photophysical properties both in solution [23] and in a solid state [22,24]. Considering the increasing current interest in the development of synthetic pyrene chemistry [25,26,27], we became interested in exploring its synthetic potential. It was found that *N*-*tert*-butylpyrene-1-carboxamide undergoes deprotonative lithiation selectively at the C2 position, which may contribute to the synthesis of new, strongly emitting pyrenyl fluorophores [28,29]. On the other hand, *N*,2,7-tri-*tert*-butylpyrene-1-carboxamide reacts with alkyllithiums in the presence of air, resulting in the products of alkylation–hydroxylation of the C9-C10 bond (K-region) [30]. 

**Scheme 1:**
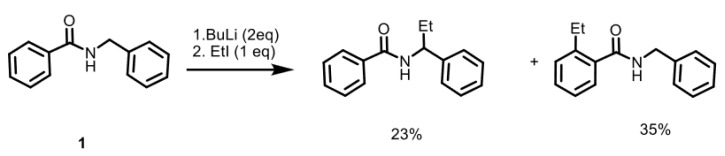
Reaction of *N*-benzylbenzamide **1** with BuLi and EtI [31].

In 2003, Murai reported that the reaction of *N*-benzylbenzamide **1** with butyllithium, followed by quenching with EtI, led to the formation of a mixture of products resulting from competitive benzylic and directed *ortho*-lithiation (Scheme 1) [31].

This finding prompted us to investigate whether a similar reaction would be possible with the pyrenyl analog of **1**, *N*-benzylpyrene 1-carboxamide **2**. Such a reaction would be of importance for tuning the fluorescent properties of pyrene amides. 

Herein, we report that **2** undergoes not only reactions analogous to those described in Scheme 1, but also unexpected cyclization via carbolithiation of the pyrene K-region, which leads to the formation of new luminescent polycyclic nitrogen compounds, bearing the *aza*-benzo[*c,d*]pyrene (azaolympicene) skeleton. We also determined basic photophysical properties (absorption and emission spectra and emission quantum yields) of selected compounds.

## 2. Results

### 2.1. Synthesis of **3**–**9**

**Scheme 2:**
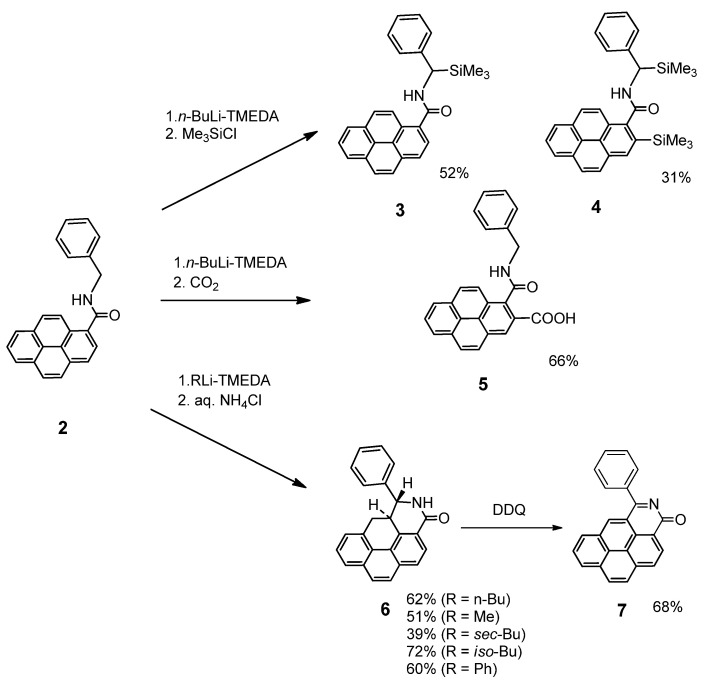
Lithiation of **2** and quenching with various electrophiles.

The lithiation reactions performed on amide **2** followed by electrophilic quenching are shown in Scheme 2.

First, we treated the solution of **2** and TMEDA in THF with *n*-BuLi (2 eq.) at –78 °C for 1 h and then quenched with TMSCl. The resulting mixture of the product silylated at the benzylic position **3** (52%) and the *bis* silylated product **4** (31%), which were easily separated by column chromatography. However, we did not observe formation of the product monosilylated at C-2. On the other hand, when CO_2_ was used as a quencher, acid **5**, resulting from the lithiation of C-2, was isolated in a 66% yield.

The observed dependence of the reaction outcome on the electrophile used prompted us to check the quenching with the simplest electrophile, H^+^. However, surprisingly, the quenching of the lithiated **2** with an aqueous solution of NH_4_Cl did not lead to the recovery of **2**; instead, the product of the intramolecular carbolithiation of pyrene K-region **6** was isolated with a 62% yield. Other organolithiums were also found to be efficient (Scheme 2), and the highest isolated yield of **6** (72%) was obtained with the use of iso-BuLi. It should be mentioned that even less reactive MeLi and PhLi produced **5** in acceptable yields (51% and 60%, respectively).

**Figure 1:**
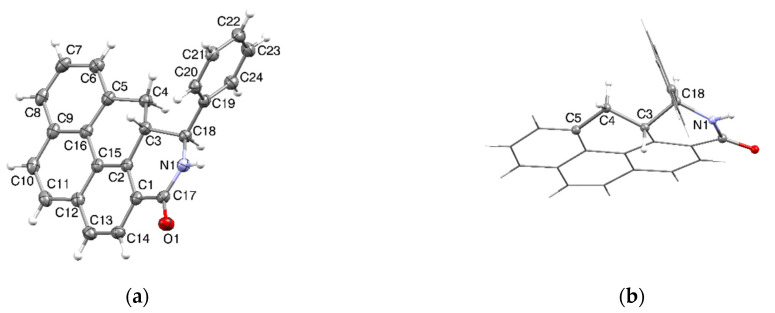
(**a**) Compound **6** in ORTEP representation, with atomic displacement parameters at 50% probability level; H atom labels omitted for clarity; (**b**) highlighted molecular conformation and the *trans* (*e,e*)-junction of the non-aromatic rings with axial hydrogens at the stereogenic carbon atoms C3 and C4; phenyl ring projecting from the image plane towards the observer.

The ^1^H NMR spectrum of compound **6** revealed that the *trans* (*e,e*)-junction of the non-aromatic rings with axial hydrogens was formed at the two stereogenic carbon atoms (corresponding coupling constant *^3^J_H-H_* = 12 Hz). This was also confirmed by single-crystal X-ray structure determination (Figure 1).

**Figure 2:**
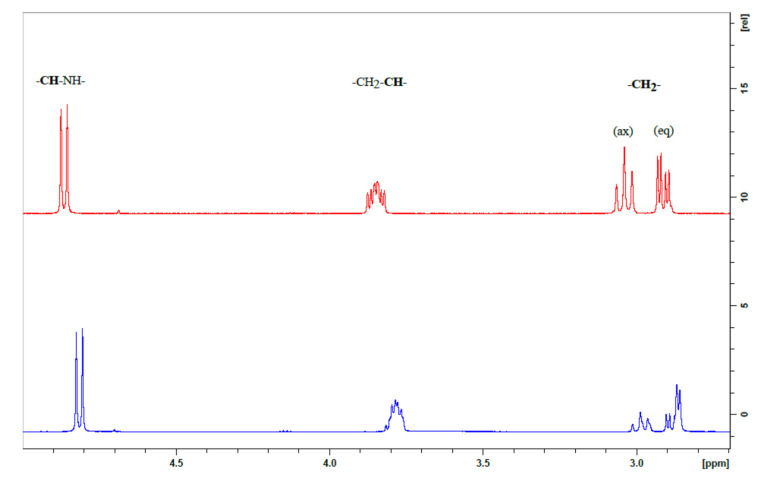
The aliphatic protons region of the ^1^H NMR spectra of **6** (top) and partly deuterated **6** (bottom).

**Figure 3:**
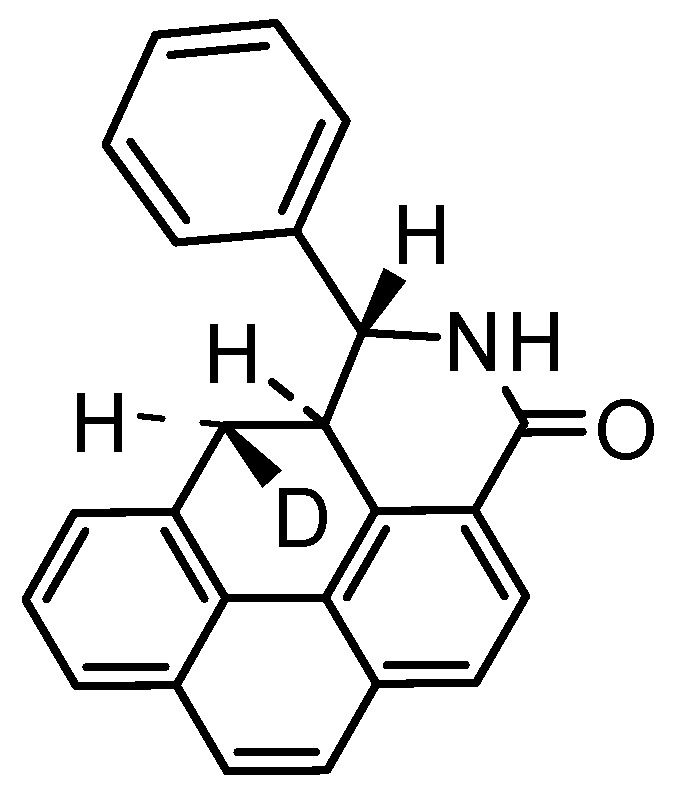
The molecular structure of **6-d**.

When using a solution of ND_4_Cl in D_2_O as a quencher, we obtained **6-d**, exhibiting ~60% monodeuteration of the CH_2_ group. The ^1^H NMR spectrum (Figure 2) revealed that the introduced isotope occupied the axial position (Figure 3). Therefore, the reaction led to the formation of three stereogenic centers in a highly stereoselective manner.

**Scheme 3:**
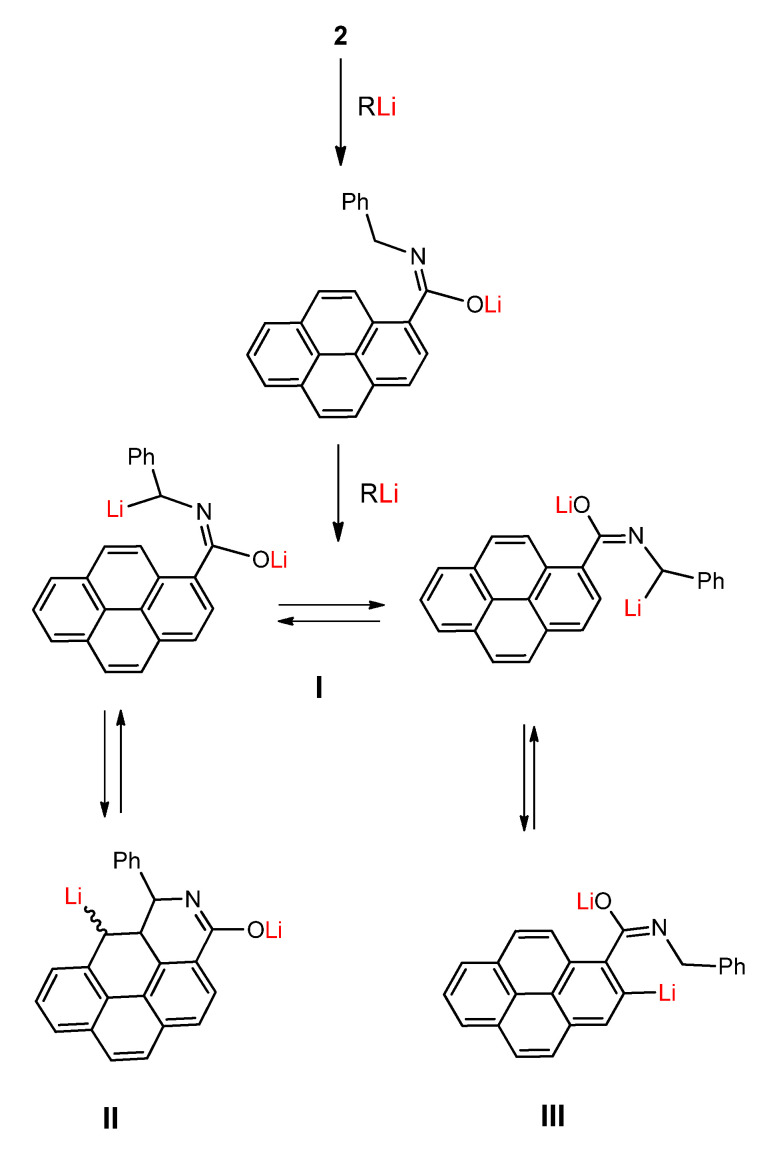
Double lithiation of **2**.

The above results can be explained by assuming that the reaction of compound **2** with BuLi-TMEDA leads to double (N, C) deprotonation, being similar to that observed in the lithiation of *N*-benzylthioamides [32], but resulting in an equilibrium mixture of dilithiated species **I-III** (Scheme 3).

It is most likely that **II** is the dominant component and that its fast protonation leads to the formation of compound **6**. However, **II** could be less reactive (possibly for steric reasons) to other electrophiles, opening the pathways to **3** and **5**. It may also be involved in the deprotonation of monolithio compounds to form **I**, which might explain the only partly deuteration observed in the quenching with D_2_O. The double silylation that leads to compound **4** can be explained by assuming that some lithiation takes place after the addition of TMSCl, which can significantly modify its course [33].

To our knowledge, the previously reported examples of intramolecular carbolithiation of aromatic *N*-benzylamides are limited to sterically hindered **tertiary** substrates [34]. Therefore, the transformation **6**→**7** constitutes the first example of such a reaction with a **secondary** amide.

Pyrene readily undergoes aromatic electrophilic substitution reactions (positions 1,3,6, and 8 are particularly reactive) [26,27]. Moreover, the introduction of a secondary amide group at position 1 allowed the lithiation of position 2 [28] and a nucleophilic attack of RLi on the C9-C10 bond (K region) [30]. In this study, we have shown that intramolecular nucleophilic addition to this region with the lithiated *N*-benzyl fragment is also possible.

**Scheme 4:**
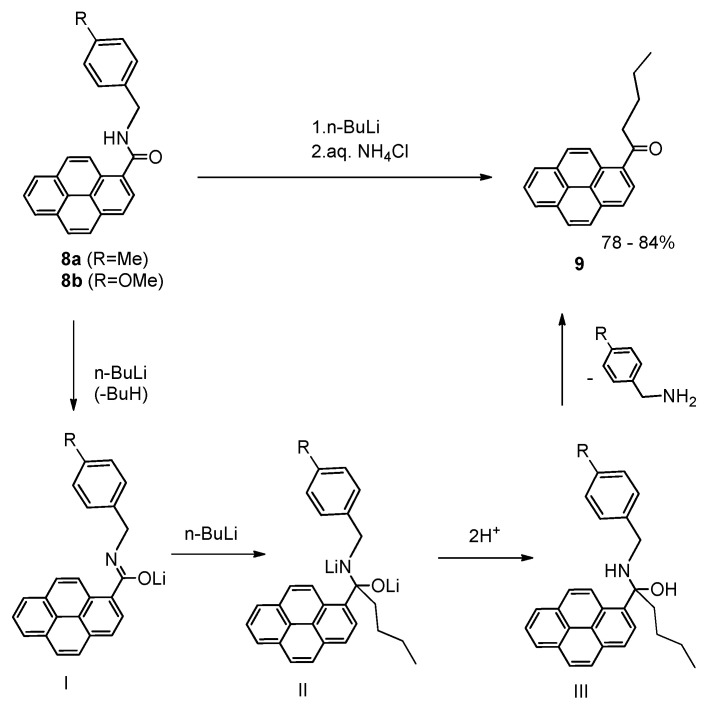
Lithiation of **8a** and **8b**.

Attempts to extend the reaction scope to the amides substituted in the phenyl ring **8a** and **8b** gave an unexpected result. The reaction of both compounds with BuLi gave 1-valerylpyrene (**9**) in good yield (Scheme 4). This compound was formed regardless of whether TMSCl, or CO_2_ followed by NH_4_Cl_aq._, or only NH_4_Cl_aq_. were used in the quenching step. The plausible reaction mechanism is presented in Scheme 4.

To our knowledge, this course of reaction was unprecedented. Until now, the synthesis of ketones from secondary amides and organolithium reagents required prior activation of amide with triflic anhydride [35] and in situ generation of organocerium species (addition of CeCl_3_) [36].

The reasons why a slight change in the structure of the benzyl group caused the observed complete change in the course of the reaction are unclear. Further research would be required to explain them, as well as to explore the possibility of extending the reaction scope.

### 2.2. Dehydrogenation of **6**

Compound **6** contained a previously unknown partially hydrogenated azaolympicene skeleton. As nitrogen-containing polycyclic aromatic compounds have been gaining continuously increasing interest [37,38], we decided to dehydrogenate this compound to the fully conjugated compound **7**. We found that this compound is formed in a 68% yield from the reaction of **6** with DDQ [39] in the refluxing dioxane.

### 2.3. Photophysical Properties of **2**, **6** and **7**

**Figure 4:**
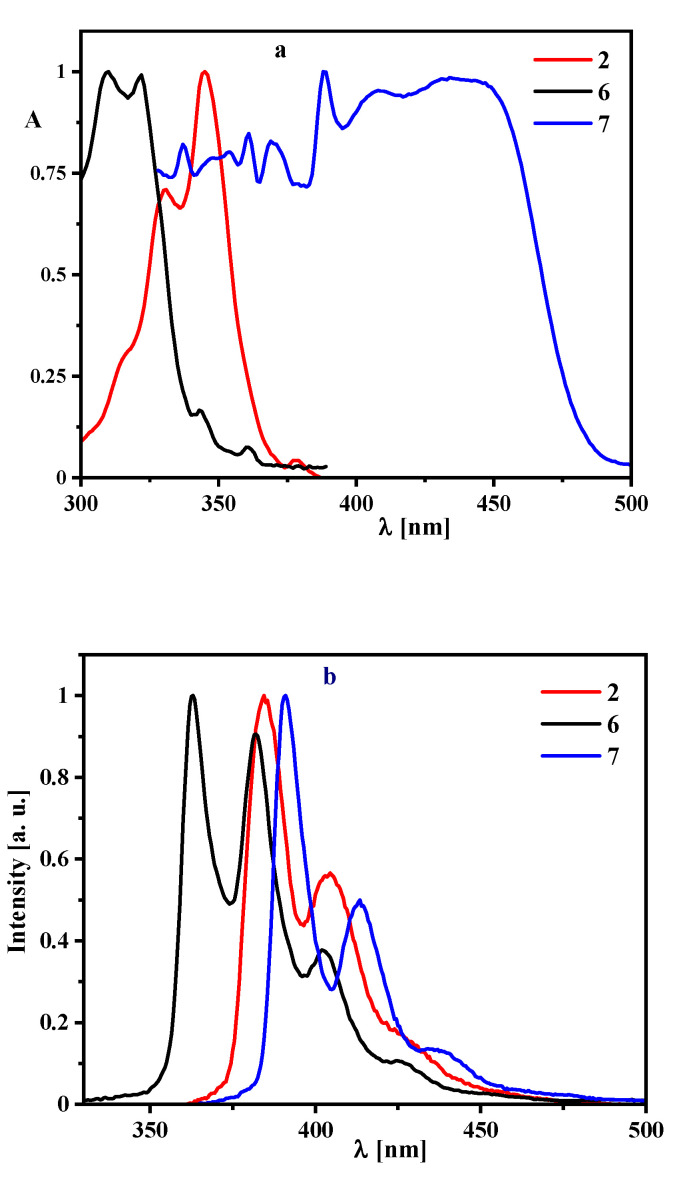
Normalized absorption (**a**) and emission (**b**) spectra of **2**, **6**, and **7** in chloroform, C = 5 × 10^−6^ M. Excitation at 345 nm (**2**), 343 nm (**6**) and 354 nm (**7**).

Ring formation involving the amide group was expected to modify the optical properties of the pyrene-1-carboxamide fluorophore. This assumption was verified by measuring the electronic absorption, emission spectra and emission quantum yields of compounds **6** and **7** and, for comparison, those of **2**. Measurements were carried out in dilute chloroform solutions (C = 10^−6^–10^−5^ M). The spectra are shown in Figure 4 and the spectroscopic data are presented in Table 1.

**Figure 5:**
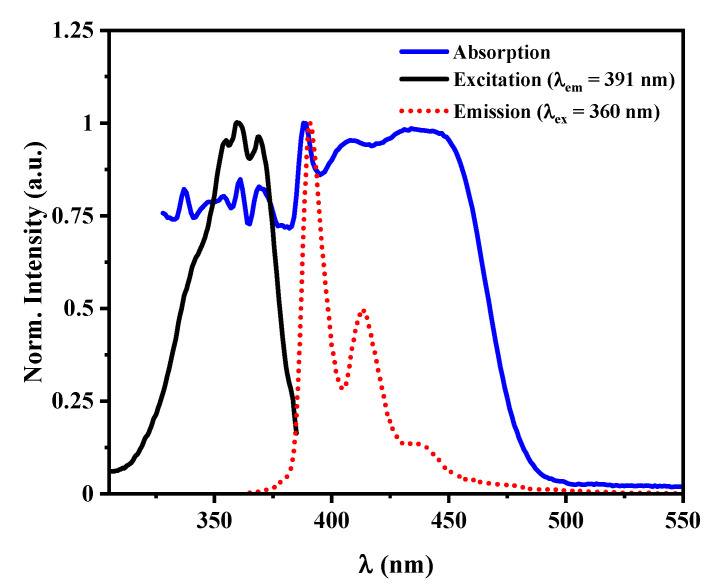
Comparison of normalized absorption, excitation, and emission spectra of **7** in chloroform, C = 5 × 10^−6^ M.

Although the absorption spectra of compounds **2** and **6** can be interpreted as resulting from the formation of locally excited (LE) states of pyrene and phenanthrene fluorophores, respectively, the broad absorption band of compound **7** can be explained by the formation of two distinct excited states [40]. However, due to the well-resolved vibronic structures, the emission spectra of the three compounds can be assigned to LE states. This implies that the second excited state of **7** is nonemissive (dark), which was confirmed by comparing the absorption and excitation spectra of this compound (Figure 5).

**Figure 6:**
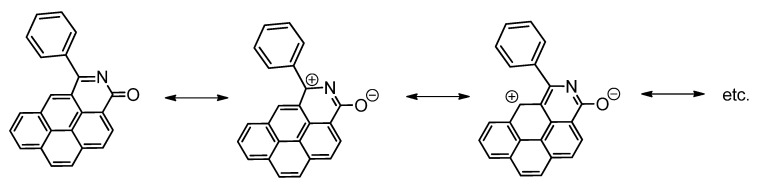
Resonance structures of **7**.

We speculate that the dark excited state of **7** may be an ICT (intramolecular charge transfer) state, characterized by increased importance of dipolar structures (Figure 6). This state may be non-radiatively deactivated by a rotation of the phenyl ring.

## 3. Conclusions

Our study showed that the lithiation of **2** followed by electrophilic quenching can lead to a substitution at the benzylic and/or C-2 position, as well as to intramolecular carbolithiation of the pyrene K-region, depending on the electrophile used in the reaction. The latter pathway allows the synthesis of a novel polycyclic nitrogen system, aza-benzo[c,d]pyrene (azaolympicene). The obtained results, along with those of our previous studies [28,29,30], show the considerable potential of lithiation of pyrene amides as a tool for functionalization of the pyrene skeleton and synthesis of new fluorophores.

## 4. Materials and Methods

All reagents and solvents were purchased from Sigma-Aldrich and used without further purification. Compound **2** was prepared as described in the literature [22]. Column chromatography was carried out on silica gel 60 (0.040–0.063 mm and 230–400 mesh, Fluka). ^1^H and ^13^C NMR spectra were recorded at room temperature (291 K) in CDCl_3_ on a Bruker ARX 600 MHz spectrometer (600 MHz for ^1^H and 151 MHz for ^13^C). Chemical shifts are in ppm and coupling constants in Hz. Elemental analyses were performed in the Microanalytical Laboratory at the Faculty of Chemistry, University of Lodz.

### 4.1. Synthesis of **3**–**7**

#### 4.1.1. Lithiation–Electrophilic Quenching of N-Benzylpyrene-1-Carboxamide 2

To a stirred solution of *N*-benzylpyrene-1-carboxamides **2**, **8a** or **8b** (1 mmol) and TMEDA (430 µL, 2 mmol) in THF (30 mL) *n*-butyllithium (1.6 M in hexanes, 1.25 mL, 2 mmol) or an equivalent amount of another RLi reagent was added at −78 °C. The solution was kept at −78 °C for 1 h and an electrophile (2 mmol, or excess of CO_2_) was added. Stirring at −78 °C was continued for 1.5 h, and then the reaction mixture was warmed to RT, followed by the addition of a saturated aqueous solution of ammonium chloride (5 mL). The products **3**, **4, 6** and **9** were extracted with dichloromethane, and the extracts were dried (Na_2_SO_4_) and evaporated to dryness. The crude products were purified by column chromatography (silica gel, hexane: ethyl acetate 5:1 for **3** and **4**, CH_2_Cl_2_: ethyl acetate: hexane 5:1:7 for **6** and CHCl_3_ for **9**). The product **5** was filtered off directly from the reaction mixture, washed with methanol and dried.

*N*-(Phenyl(trimethylsilyl)methyl)pyrene-1-carboxamide (**3**). White solid (213 mg, 52%); mp = 191–192 °C. ^1^H NMR (600 MHz, CDCl_3_) δ 8.52 (d, *J* = 9.0 Hz, 1H), 8.23 (d, *J* = 2.4 Hz, 1H), 8.22 (d, *J* = 3.0 Hz, 1H), 8.19 (d, *J* = 7.8 Hz, 1H), 8.14 (d, *J* = 9.0 Hz, 2H), 8.11 (d, *J* = 7.8 Hz, 1H), 8.09 (d, *J* = 9.0 Hz, 1H), 8.05 (t, *J* = 7.8 Hz, 1H), 7.39–7.34 (m, 2H), 7.28–7.20 (m, signals of CDCl_3_ and 3H), 6.50 (d, *J* = 9.0 Hz, 1H), 5.10 (d, *J* = 9.0 Hz, 1H), 0.15 (s, 9H) ppm; ^13^C{^1^H} NMR (151 MHz, CDCl_3_) δ 169.6, 141.4, 132.3, 131.4, 131.1, 130.6, 128.5, 128.4, 126.9, 126.2, 126.1, 125.8, 125.7, 125.6, 124.6, 124.4, 124.3, 124.3, 124.2, 47.3, −3.2; Anal. calcd. for C_27_H_25_NOSi: C, 79.56; H, 6.18; N, 3.44; found: C, 79.60; H, 6.25; N, 3.41.

*N*-(Phenyl(trimethylsilyl)methyl)-2-(trimethylsilyl)pyrene-1-carboxamide (**4**). White solid (150 mg, 31%); mp = 147–149 °C. ^1^H NMR (600 MHz, CDCl_3_) δ 8.38 (s, 1H), 8.20 (t, *J* = 7.8 Hz, 2H), 8.14 (d, *J* = 9.6 Hz, 1H), 8.12 (d, *J* = 9.0 Hz, 1H), 7.38 (t, *J* = 7.8 Hz, 2H), 7.30–7.25 (m, 3H), 6.36 (d, *J* = 9.0 Hz, 1H), 5.13 (d, *J* = 9.0 Hz, 1H), 0.40 (s, 9H), 0.16 (s, 9H) ppm; ^13^C{^1^H} NMR (151 MHz, CDCl_3_) δ 171.0, 141.0, 137.8, 135.3, 131.4, 131.2, 130.8, 130.6, 128.4, 128.1, 128.0, 127.5, 127.3, 127.2, 126.4, 126.1, 125.5, 125.2, 124.7, 124.5, 124.4, 47.6, 0.24, −2.9; Anal. Calcd. for C_30_H_33_NOSi_2_: C, 75.10; H, 6.93; N, 2.92; found: C, 75.25; H, 6.99; N, 2.88.

1-Benzylcarbamoilpyrene-2-carboxylic acid (**5**). Yellow solid (251 mg, 66%); mp = 265–268 °C; ^1^H NMR (600 MHz, DMSO): δ 9.02 (t, *J* = 5.4 Hz, 1H), 8.84 (s, 1H), 8.37 (dd, *J* = 7.8 Hz, *J* = 7.2 Hz, 2H), 8.33 (d, *J* = 9.0 Hz, 1H), 8.29 (d, *J* = 9.0 Hz, 1H), 8.26 (d, *J* = 9.6 Hz, 1H), 8.17 (t, *J* = 7.8 Hz, 1H), 8.09 (d, *J* = 9.6 Hz, 1H), 7.53 (d, *J* = 7.8 Hz, 2H), 7.39 (t, *J* = 7.8 Hz, 2H), 7.29 (t, *J* = 7.2 Hz, 1H), 4.66 (d, *J* = 5.4 Hz, 2H) ppm; ^13^C{^1^H} NMR (151 MHz, DMSO): δ 168.3, 167.5, 139.4, 134.1, 131.3, 130.6, 130,2, 128.6, 128.5, 128.3, 127.8, 127.7, 127.5, 126.8, 126.1, 125.9, 125.8, 125.3, 124.8, 123.2, 43.1; Anal. Calcd. for C_25_H_17_NO_3_: C, 79.14; H, 4.52; N, 3.69; found: C, 79.11; H, 4.56; N, 3.71.

5-Phenyl-4-aza-4,5,5a,6-tetrahydro-3*H*-benzo[*c,d*]-pyren-3-one (**6**). White solid (242 mg, 72%); mp = 200–202 °C. ^1^H NMR (600 MHz, CDCl_3_) δ 8.35 (d, *J* = 8.4 Hz, 1H), 7.91 (d, *J* = 7.8 Hz, 1H), 7.85 (d, *J* = 9.0 Hz, 1H), 7.79 (d, *J* = 9.0 Hz, 1H), 7.76 (d, *J* = 8.4 Hz, 1H), 7.58–7.53 (m, 2H), 7.52–7.45 (m, 4H), 7.27 (d, *J* = 6.6 Hz, 1H), 6.08 (s, 1H), 4.87 (d, *J* = 12 Hz, 1H), 3.89–3.79 (m,1H), 3.04 (t, *J* = 15 Hz, 1H), 2.92 (dd, *J* = 6.6 Hz, 1H) ppm; ^13^C{^1^H} NMR (151 MHz, CDCl_3_) δ 165.9, 165.8, 139.2, 139.2, 135.7, 135.7, 133.6, 133.6, 132.5, 130.9, 129.2, 129.1, 129.0, 128.9, 127.7, 127.5, 127.0, 126.7, 126.7, 126.4, 126.2, 125.9, 125.7, 125.3, 124.3, 124.3, 63.1, 39.7, 30.9; Anal. Calcd. for C_24_H_17_NO: C, 85.94; H, 5.11; N, 4.18. Found: C, 85.97; H, 5.08; N, 4.21.

1-(Pyren-1-yl)pentan-1-one (**9**). Yellow solid (241 mg, 84%). ^1^H NMR (500 MHz, CDCl_3_) δ 8.86 (d, *J* = 9.0 Hz, 1H), 8.30 (d, *J* = 8.0 Hz, 1H), 8.23 (m, 2H), 8.19 (d, *J* = 9.5 Hz, 1H), 8.16 (d, *J* = 8.0 Hz, 1H), 8.15 (d, *J* = 9.0 Hz, 1H), 8.05 (m, 2H), 3.21 (t, *J* = 7.5 Hz, 2H), 1.85 (m, 2H), 1.50 (m, 2H), 0,99 (t, *J* = 7.5 Hz, 3H); ^13^C{^1^H} NMR (125 MHz, CDCl_3_) δ 205.4, 133.5, 132.8, 131.0, 130.5, 129.4, 129.3, 129.1, 127.0, 126.3, 126.1, 125.9, 125.8, 124.9, 124.7, 124.3, 123.9, 42.3, 27.0, 22.5, 13.9; Anal. Calcd. for C_21_H_18_O: C, 88.08; H, 6.34. Found: C, 88.14; H, 6.37.

#### 4.1.2. Dehydrogenation of Compound **6**

To a solution of **6** (200 mg, 0.6 mmol) in dioxane, 10 mL of DDQ (340 mg, 1.5 mmol) was added. The mixture was stirred at reflux for 20 h. After cooling to RT, water (100 mL) was added, and the mixture was extracted with dichloromethane (5 × 50 mL). The combined organic solutions were dried over anhydrous MgSO_4_. After evaporating the solvent, the crude product was purified by column chromatography (silica gel and dichloromethane).

5-Phenyl-4-aza-4,5,5a,6-tetrahydro-3*H*-benzo[*c,d*]-pyren-3-one (**6**). White solid (242 mg, 72%); mp = 200–202 °C. ^1^H NMR (600 MHz, CDCl_3_) δ 8.35 (d, *J* = 8.4 Hz, 1H), 7.91 (d, *J* = 7.8 Hz, 1H), 7.85 (d, *J* = 9.0 Hz, 1H), 7.79 (d, *J* = 9.0 Hz, 1H), 7.76 (d, *J* = 8.4 Hz, 1H), 7.58–7.53 (m, 2H), 7.52–7.45 (m, 4H), 7.27 (d, *J* = 6.6 Hz, 1H), 6.08 (s, 1H), 4.87 (d, *J* = 12 Hz, 1H), 3.89–3.79 (m,1H), 3.04 (t, *J* = 15 Hz, 1H), 2.92 (dd, *J* = 6.6 Hz, 1H) ppm; ^13^C{^1^H} NMR (151 MHz, CDCl_3_) δ 165.9, 165.8, 139.2, 139.2, 135.7, 135.7, 133.6, 133.6, 132.5, 130.9, 129.2, 129.1, 129.0, 128.9, 127.7, 127.5, 127.0, 126.7, 126.7, 126.4, 126.2, 125.9, 125.7, 125.3, 124.3, 124.3, 63.1, 39.7, 30.9; Anal. Calcd. for C_24_H_17_NO: C, 85.94; H, 5.11; N, 4.18. Found: C, 85.97; H, 5.08; N, 4.21.

#### 4.1.3. Synthesis of Amides **8a**-**b**

4-methyl- or 4-methoxybenzyl isocyanate (1.1 mmol) and TfOH (348 μL, 4 mmol) were added to a solution of pyrene (202 mg, 1 mmol) in CH_2_Cl_2_ (10 mL) at room temperature. After stirring for 10 min, the reaction mixture was poured into ice water (50 mL) and extracted several times with CH_2_Cl_2_. The combined extracts were dried over anhydrous Na_2_SO_4_ and evaporated. Flash chromatography (silica gel/CH_2_Cl_2_) afforded pure products.

*N*-(4-methylbenzyl)pyrene-1-carboxamide (**8a**). White solid (311 mg, 89%). ^1^H NMR (500 MHz, DMSO) δ 9.28 (d, *J* = 9.5 Hz, 1H), 8.42 (d, *J* = 8.0 Hz, 1H), 8.27 (m, 2H), 8.22 (d, *J* = 8.0 Hz, 1H), 8.16 (m, 3H), 8.06 (t, *J* = 7.5 Hz, 1H), 7.37 (d, *J* = 8.0 Hz, 2H), 7.18 (d, *J* = 7.5 Hz, 2H), 3.99 (s, 2H), 2.89 (s, 3H); ^13^C{^1^H} NMR (125 MHz, DMSO) δ 171.6, 137.0, 134.8, 133.5, 130.9, 130.8, 130.2, 128.9, 128.5, 128.4, 127.4, 127.4, 127.3, 126.8, 126.7, 126.1, 125.1, 124.9, 124.2, 124.0, 123.9, 42.5, 20.7; Anal. Calcd. for C_25_H_19_NO: C, 85.93; H, 5.48; N, 4.01. Found: C, 85.99; H, 5.56; N, 3.95.

*N*-(4-methoxybenzyl)pyrene-1-carboxamide (**8b**). White solid (318 mg, 87%). ^1^H NMR (500 MHz, DMSO) δ 9.32 (d, *J* = 9.5 Hz, 1H), 8,76 (s, 1H), 8.44 (d, *J* = 8.0 Hz, 1H), 8.25 (m, 3H), 8.15 (m, 2H), 8.16 (m, 3H), 8.05 (t, *J* = 8.0 Hz, 1H), 7.46 (d, *J* = 8,5 Hz, 2H), 6.91 (d, *J* = 8.5 Hz, 2H), 4.03 (s, 2H), 3.71 (s, 3H); ^13^C{^1^H} NMR (125 MHz, DMSO) δ 172.8, 159.5, 136.2, 131.3, 131.2, 130.8, 130.7, 128.7, 128.2, 127.9, 127.8, 127.7, 127.4, 127.16, 126.5, 125.5, 125.3, 124.7, 124.6, 124.5, 114.3, 55.5, 42.4; Anal. Calcd. for C_25_H_19_NO_2_: C, 82.17; H, 5.24; N, 3.83. Found: C, 82.26; H, 5.19; N, 3.75.

All spectra are given in the Supplementary Materials. 

### 4.2. UV/Vis Measurements

The electronic absorption spectra were obtained using a PerkinElmer Lambda 45 UV/VIS spectrometer, and corrected emission spectra were obtained using a PerkinElmer LS-55 fluorescence spectrometer. The emission quantum yields were determined using a solution of quinine sulfate in 0.5 M sulfuric acid as a reference (ΦF = 0.546) [41].

### 4.3. X-ray Diffraction Measurement

Crystals of **6** suitable for the single-crystal X-ray diffraction study were grown from DCM/hexane. The X-ray intensity data were measured on an Agilent Supernova 4 circle diffractometer system equipped with a copper (CuKα) microsource and an Atlas CCD detector. The data were collected and integrated with CrysAlis171 software (version 1.171.38.43d). Data were corrected for absorption effects using the multi-scan method CrysAlis171 software (version 1.171.38.43d) Agilent Technologies, Oxfordshire, UK. 

The sample’s low temperature was maintained by keeping it in the cold nitrogen stream, using Oxford Cryosystems cooling devices.

The structure was solved by direct methods using SXELXS [42] and refined by the full-matrix least squares procedure with SHELXL [40] within an OLEX2 [43] graphical interface. Figures were produced with Mercury_3.10 [44] software. 

All H atoms were visible in the residual density map, but were added geometrically and refined mostly in riding approximation.

Detailed information about the data processing, structure solution, and refinement is presented in Table S1.

## Figures and Tables

**Table 1:** Photophysical data of compounds **2**, **6** and **7**.

Compound	Absorption λ_max_ [nm]/ε [M^−1^·cm^−1^]	Emission [nm]	Φ_F_
**2**	331/31,620, 345/44,560, 379/1900	385, 404	0.32
**6**	310/31,100, 322/30860, 343/5180, 360/2340	363, 382, 402, 425	0.36
**7**	337/20,200, 354/19,740, 361/20,860, 369/20,380, 389/24,580, 408/23,460, 434/24,240 *	391, 413, 438	0.30

* Broad and poorly resolved spectrum.

## Data Availability

The structure of **6** has been deposited with the CCDC, deposition number 2141841.

## Supplementary Materials

The following supporting information can be downloaded at https://www.mdpi.com/article/10.3390/molecules27123930/s1: a PDF file of the ^1^H NMR and ^13^C NMR spectra of all reported compounds and a crystal structure report for compound **6**.

## Sample Availability

Samples of the compounds are available from the authors.
