# Supplementary material for: Electrophile-Dependent Reactivity of Lithiated N-Benzylpyrene-1-Carboxamide
Source: Molecules. 2022 Jun 19;27(12):3930. doi: 10.3390/molecules27123930 (PMC9227622; doi:10.3390/molecules27123930)
Supplement: Supplementary file 1 [file molecules-27-03930-s001.zip › molecules-1690846-supplementary.pdf]

Supplementary Information

Electrophile-dependent reactivity of lithiated *N*-benzylpyrene-1-carboxamide

Magdalena Ciechańska, Anna Wrona-Piotrowicz, Karolina Koprowska, Anna Makal, Janusz Zakrzewski

1. X-ray diffraction data for **6**……………………………………………………….……..2
2. ^{1}H and ^{13}C NMR spectra of **3 - 9**…………………..……………………………………...4

Table S1. X-ray diffraction data for **6**

**Compound**	**6**
Empirical formula	$C_{24} H_{17} N_1 O_1$
Formula weight	335.38
Crystal system	monoclinic
Space group	C 2/c
a/Å	25.8302(6)
b/Å	4.71042(11)
c/Å	27.0073(8)
α/°	90
β/°	93.353(2)
γ/°	90
Volume/$Å^3$	3280.39(15)
Z / Z'	8 / 1
μ/mm^{-1}	0.645
Max. transmission	1.000
Min. transmission	0.645
Absorption correction	gaussian
Crystal color	colorless
$\rho_{calc} mg/mm^3$	1.358
Crystal habit	needle
F(000)	1408
Crystal size/mm	0.41
	0.08
	0.03
R_{int}	0.022
R_{sigma}	0.021
Index ranges	
h	-29 : 31
k	-5 : 3
l	-33 : 32
Reflections collected	10475
2Θ range for data collection	73.934
	3.278
Temperature/K	120.1(1)
X-ray wavelength/Å	1.54184
Independent reflections I > 2 σ(I)	2866
Independent reflections	3253
Largest diff. peak/hole /e $Å^{-3}$	0.26 ; -0.22
Goodness-of-fit on F^2	1.036
Parameters	238
Data	3253
Restraints	0
R1 all data	0.044
R1 [I>=2σ (I)]	0.038
wR2 [I>=2σ (I)]	0.103
wR2 all data	0.108

Z' - indicates the number of crystallographically independent molecules

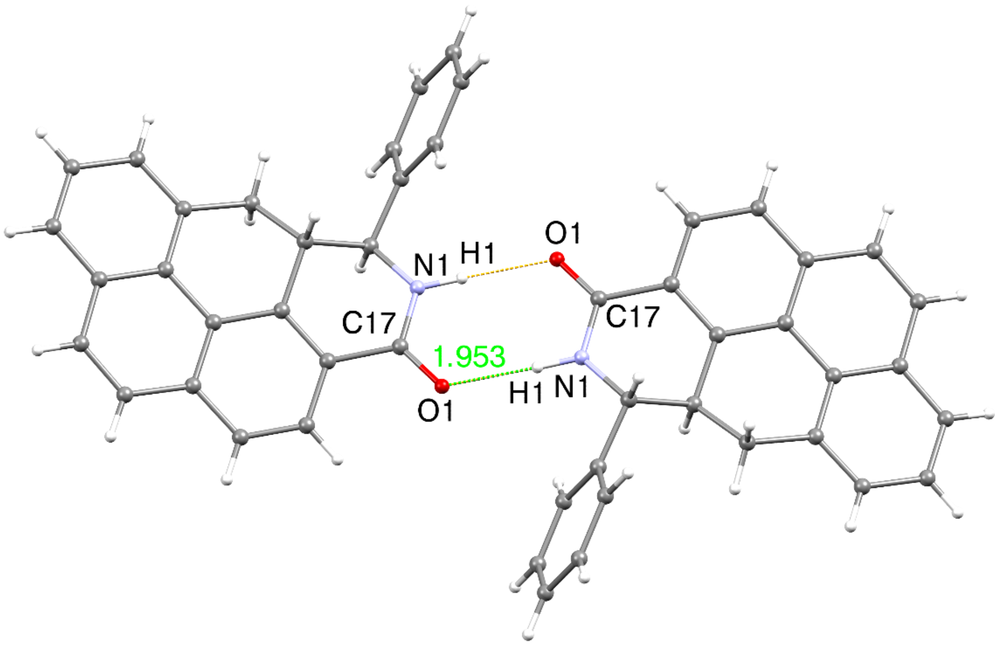

Figure S1. A set of strong N – H … O hydrogen bonds, connecting two molecules of 6 related by center of inversion. The crystal structure of **6** is composed of the stacks of such H-bonded dimers in [010] direction.

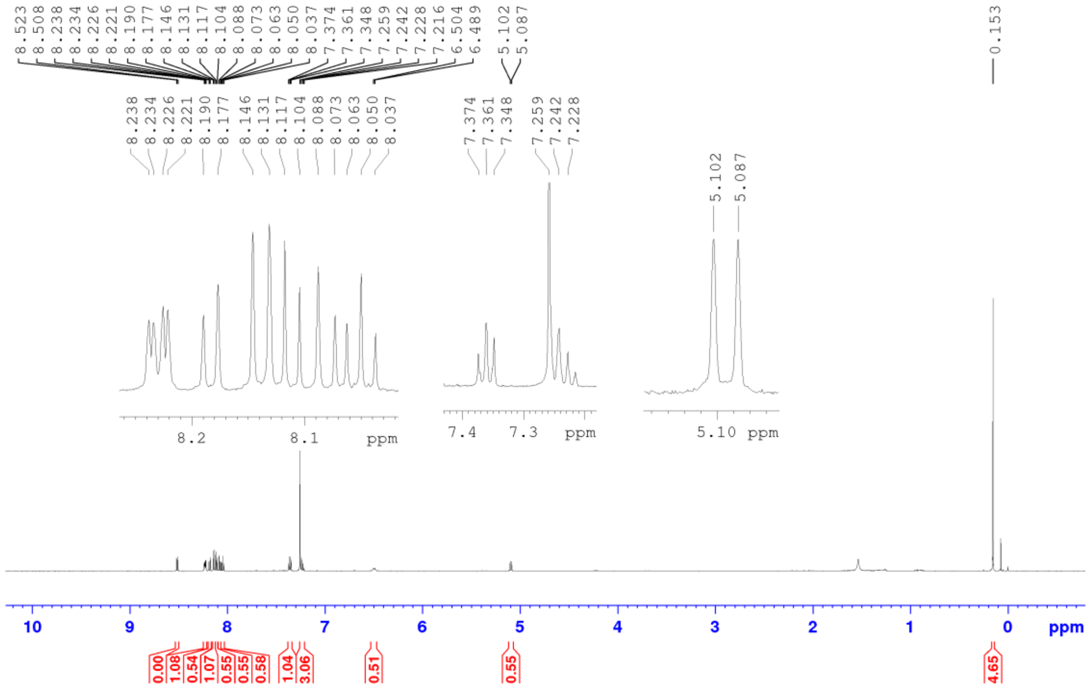

Figure S2. ^{1}H NMR spectrum of compound **3** (600 MHz, $CDCl_3$, room temperature).

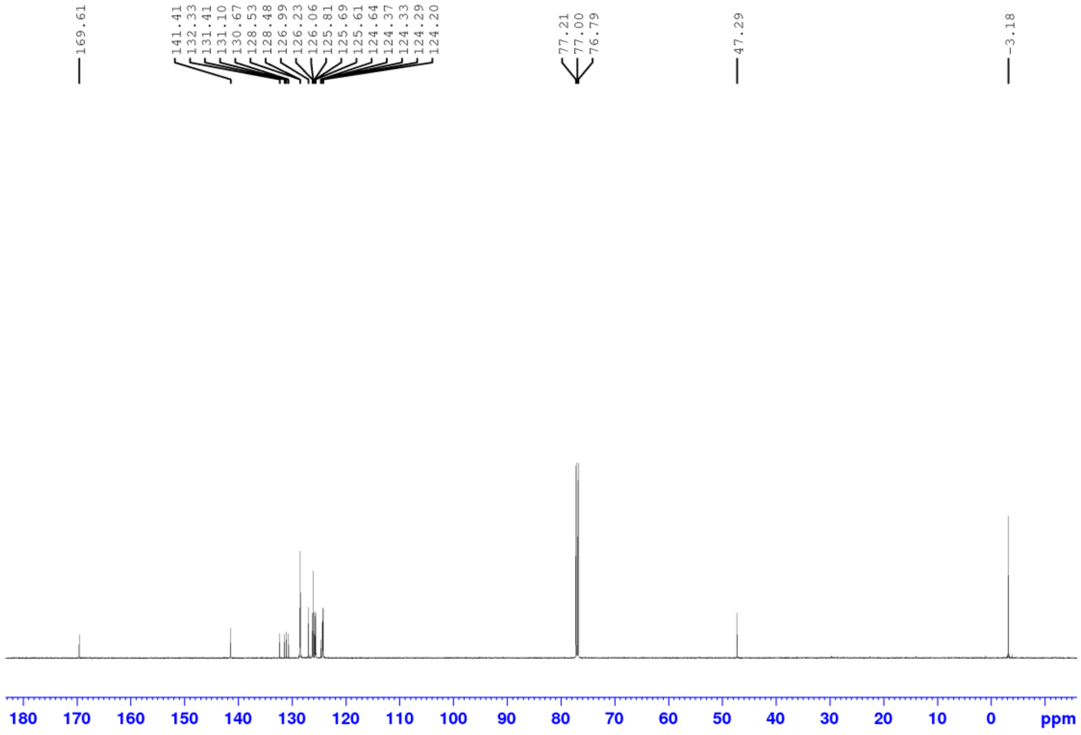

Figure S3. ^{13}C NMR spectrum of compound **3** (150 MHz, $CDCl_3$, room temperature).

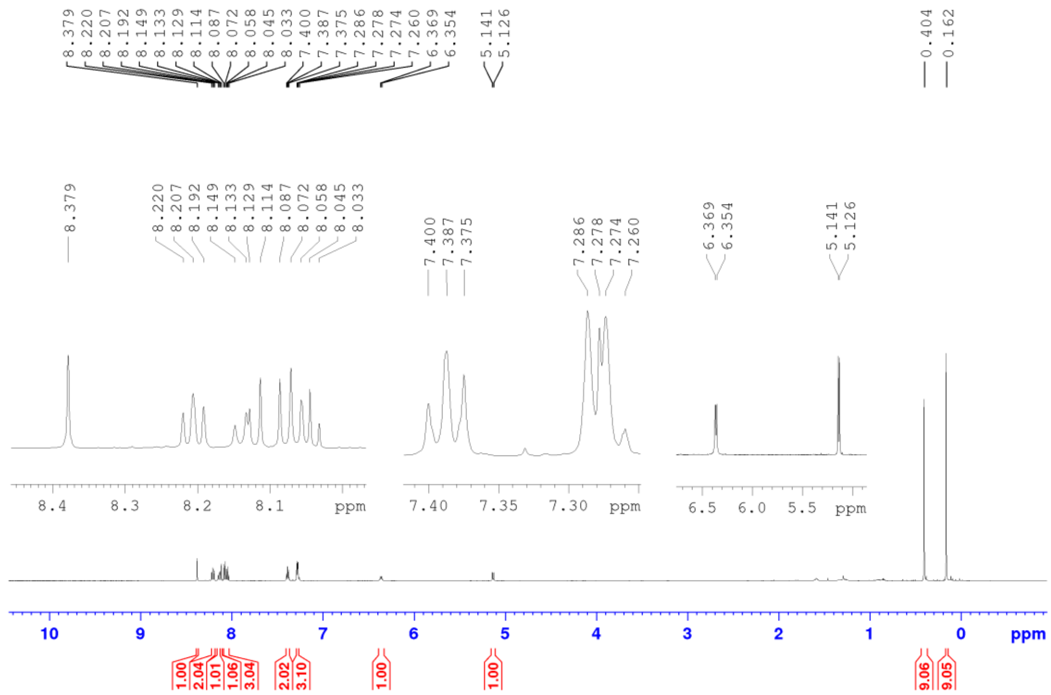

Figure S4. ^{1}H NMR spectrum of compound **4** (600 MHz, $CDCl_3$, room temperature).

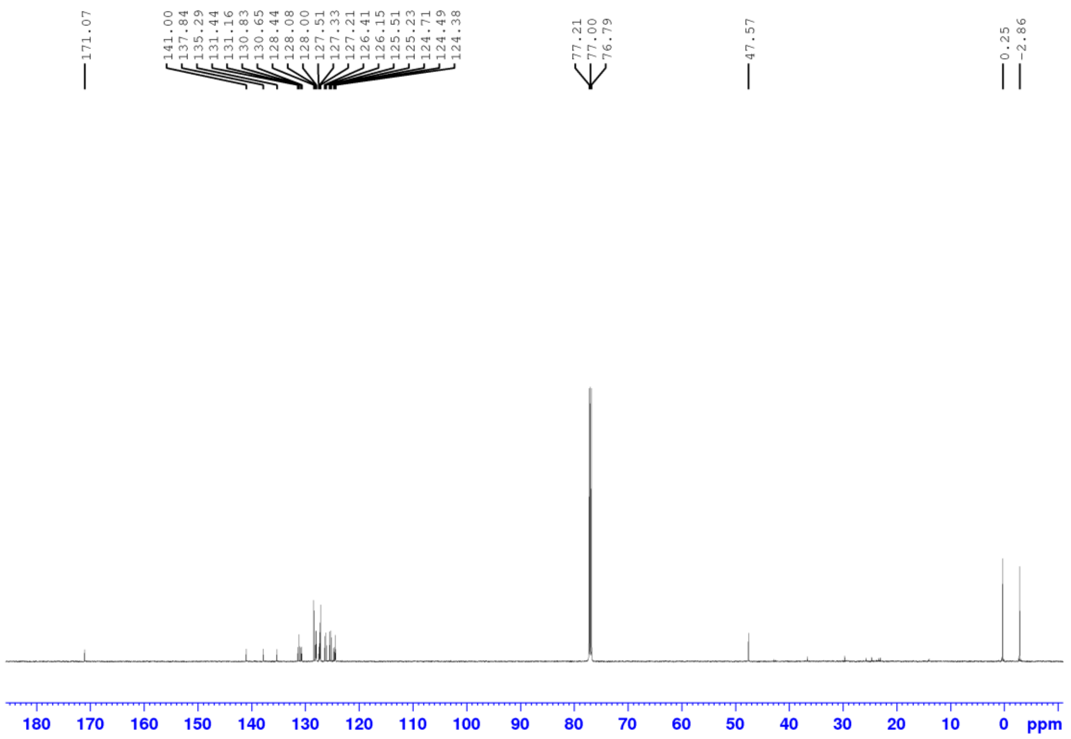

Figure S5. ^{13}C NMR spectrum of compound **4** (150 MHz, $CDCl_3$, room temperature).

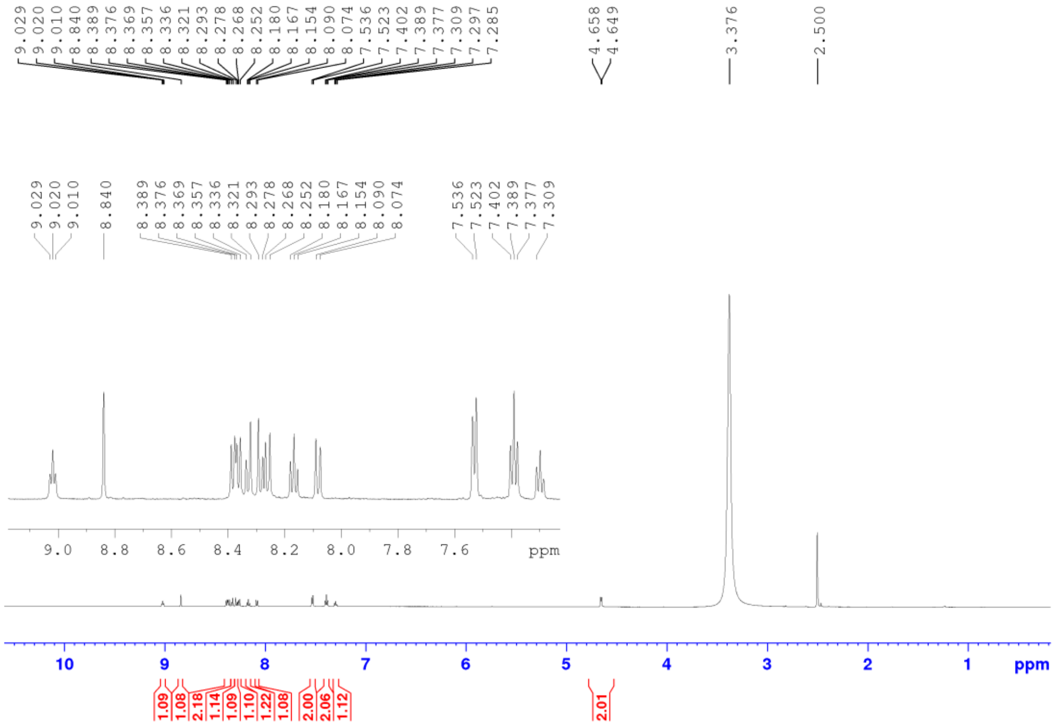

Figure S6. ^{1}H NMR spectrum of compound **5** (600 MHz, DMSO, room temperature).

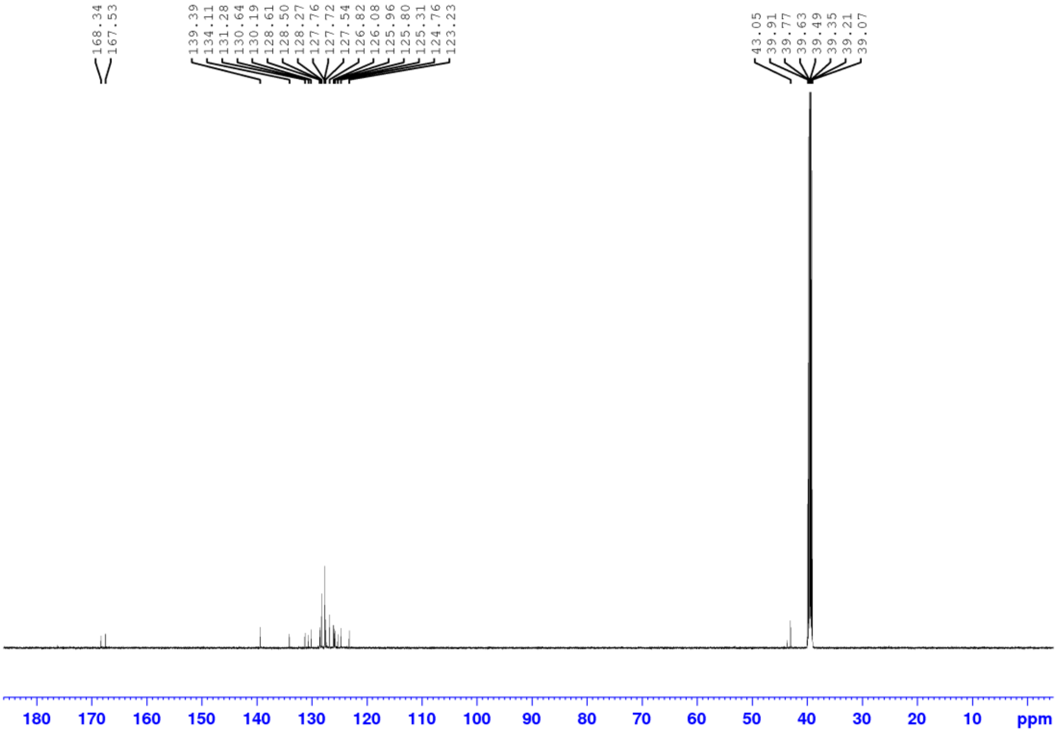

Figure S7. ^{13}C NMR spectrum of compound **5** (150 MHz, DMSO, room temperature).

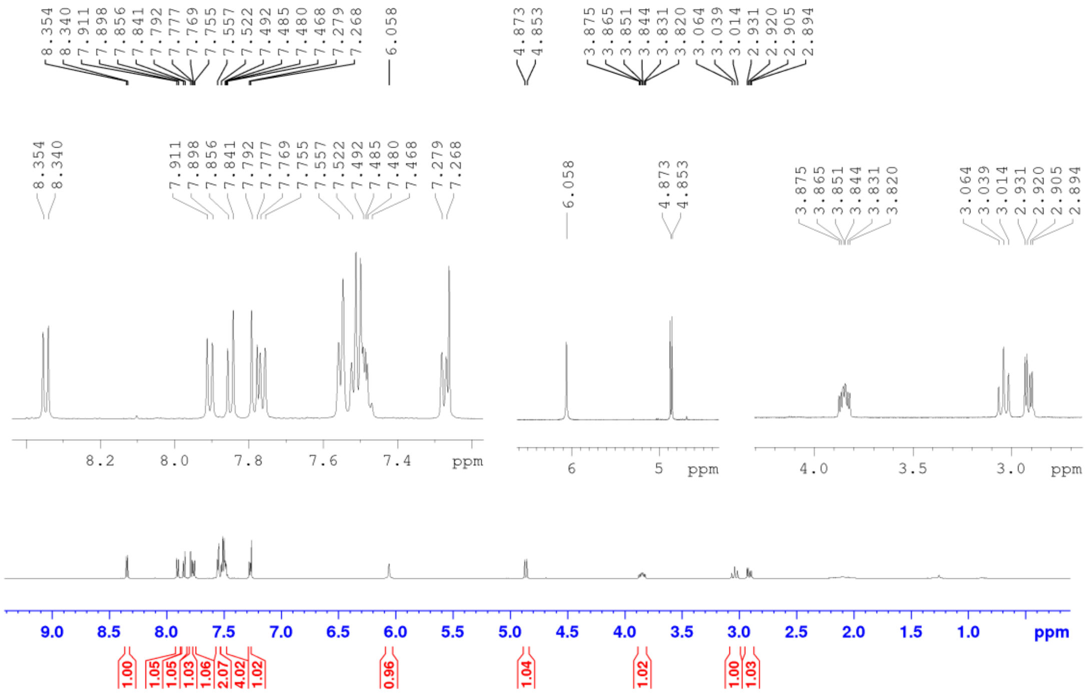

Figure S8. ^{1}H NMR spectrum of compound **6** (600 MHz, $CDCl_3$, room temperature).

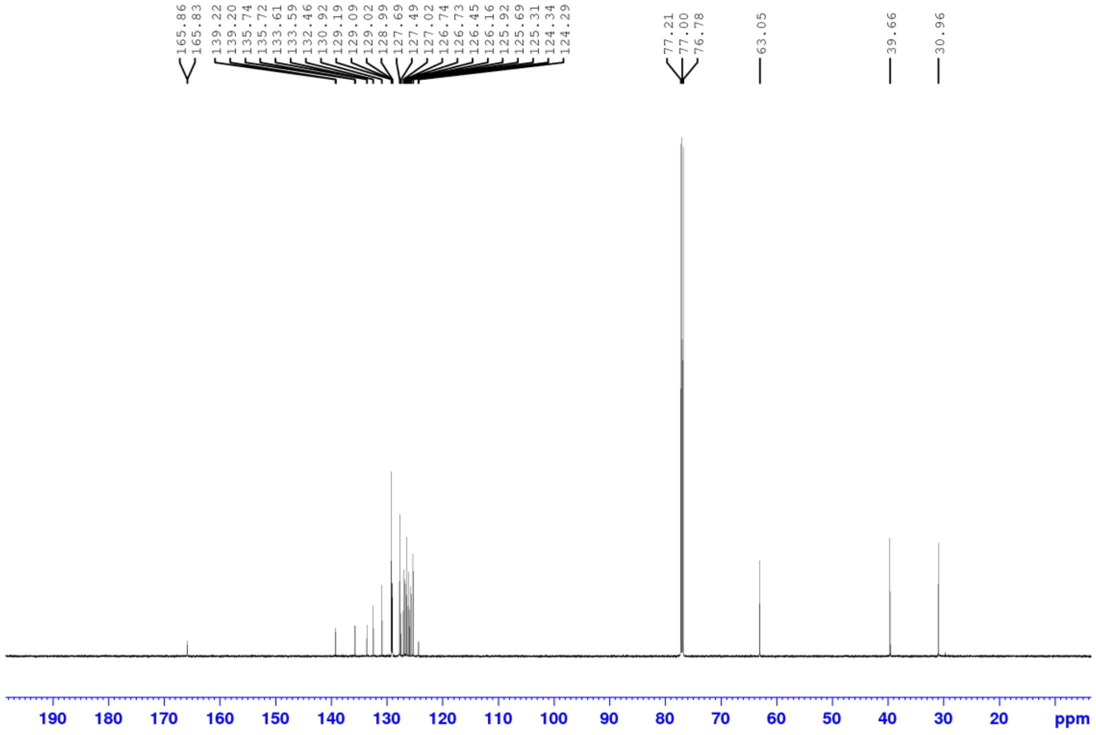

Figure S9. ^{13}C NMR spectrum of compound **6** (150 MHz, $CDCl_3$, room temperature).

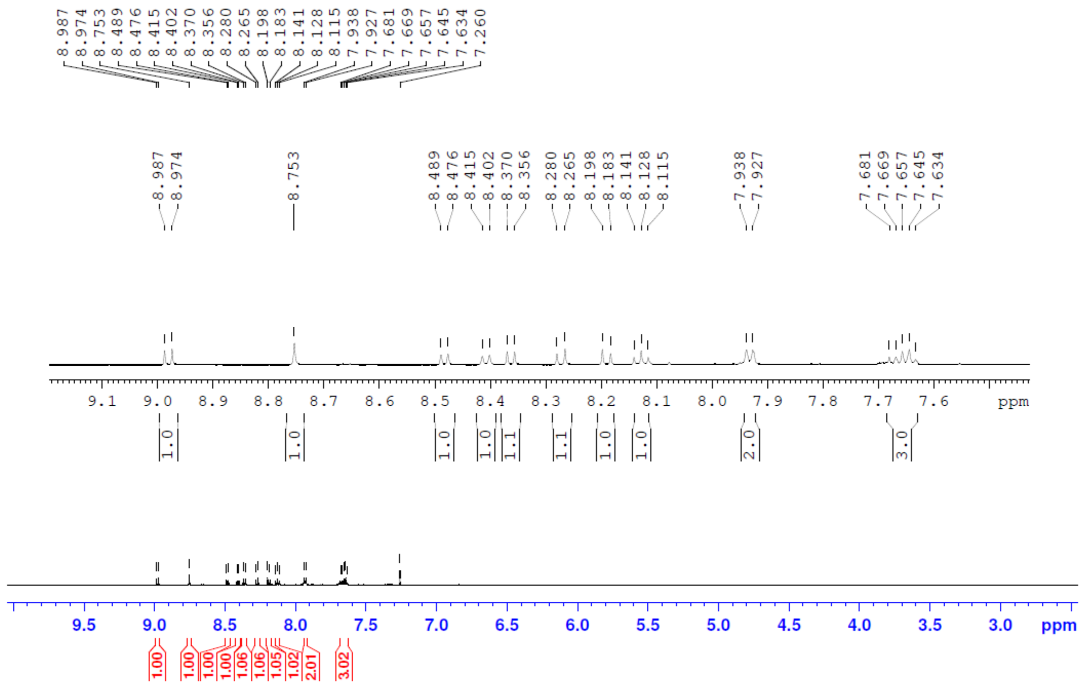

Figure S10. ^{1}H NMR spectrum of compound **7** (600 MHz, $CDCl_3$, room temperature).

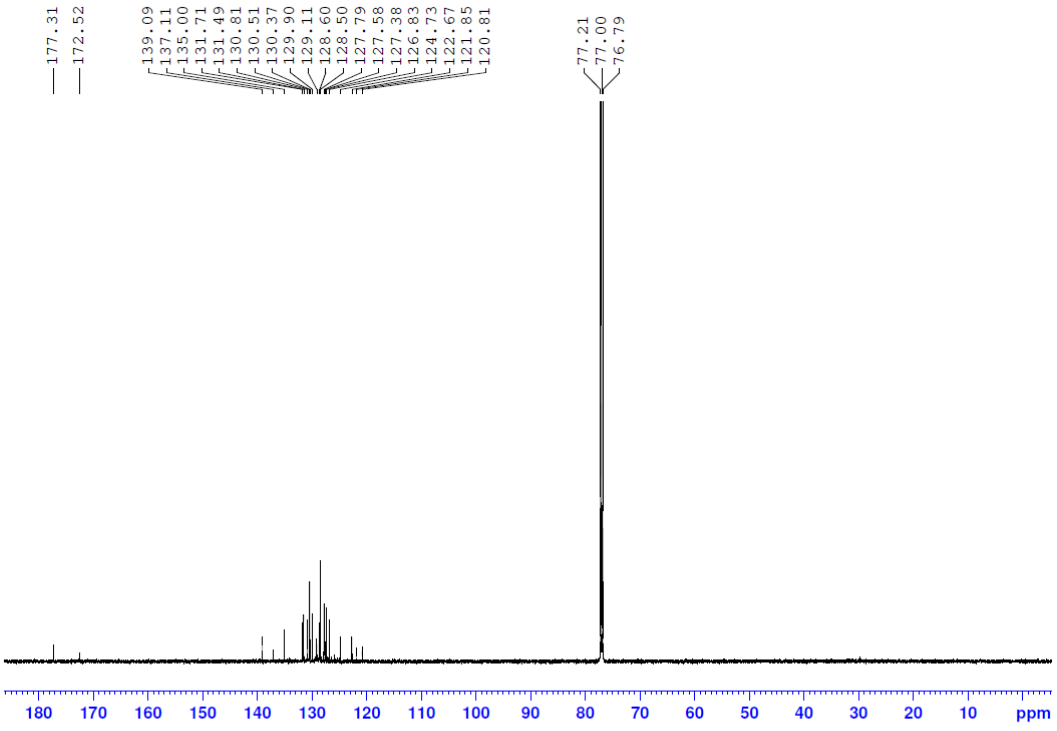

Figure S11. ^{13}C NMR spectrum of compound **7** (150 MHz, $CDCl_3$, room temperature).

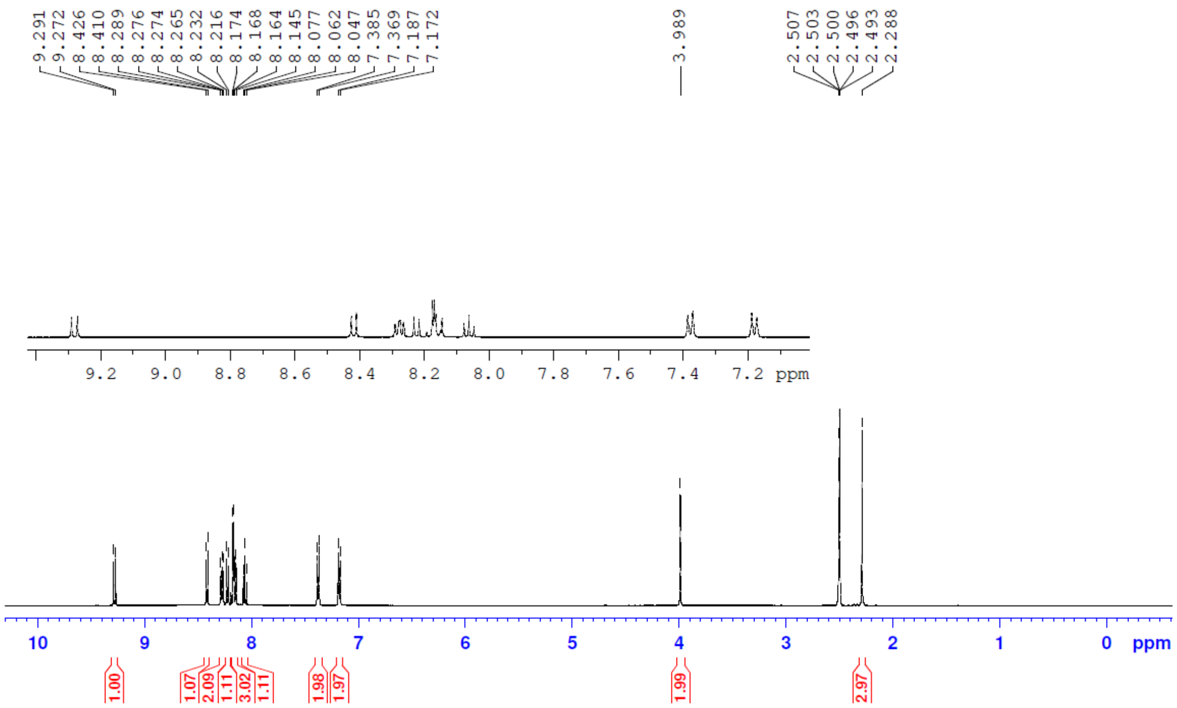

Figure S12. ^{1}H NMR spectrum of compound **8a** (500 MHz, DMSO, room temperature).

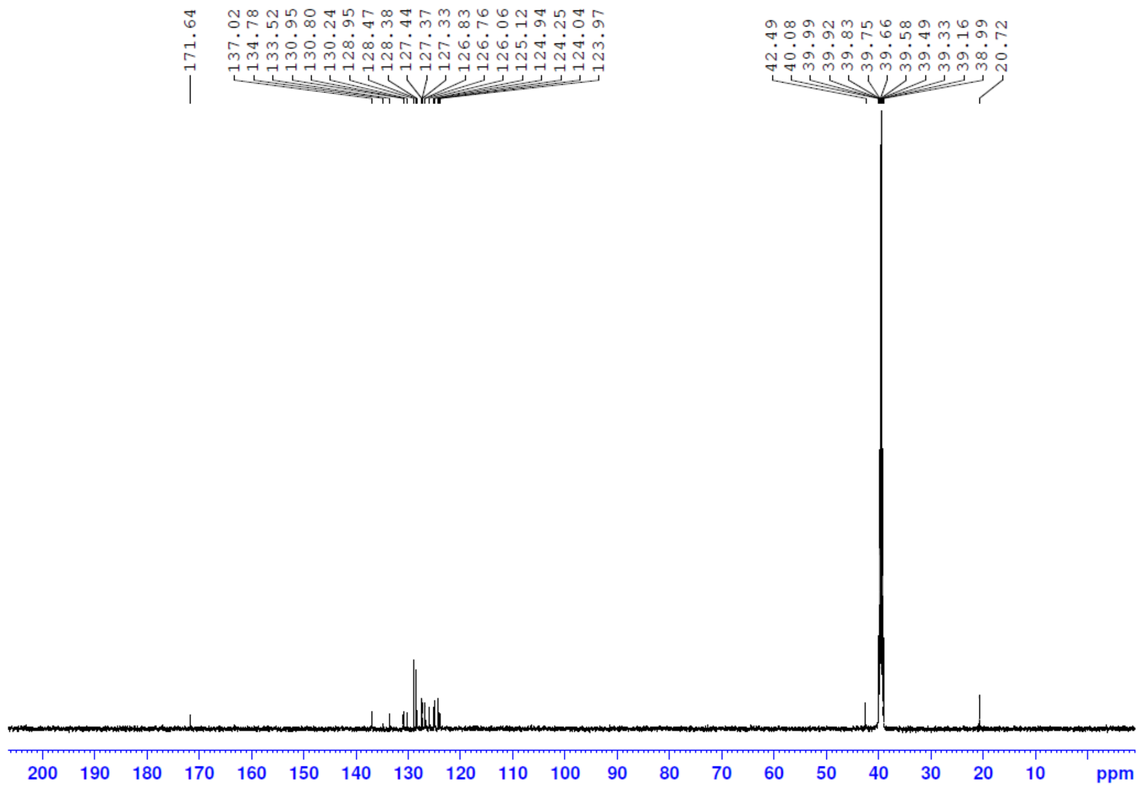

Figure S13. ^{13}C NMR spectrum of compound **8a** (125 MHz, DMSO, room temperature).

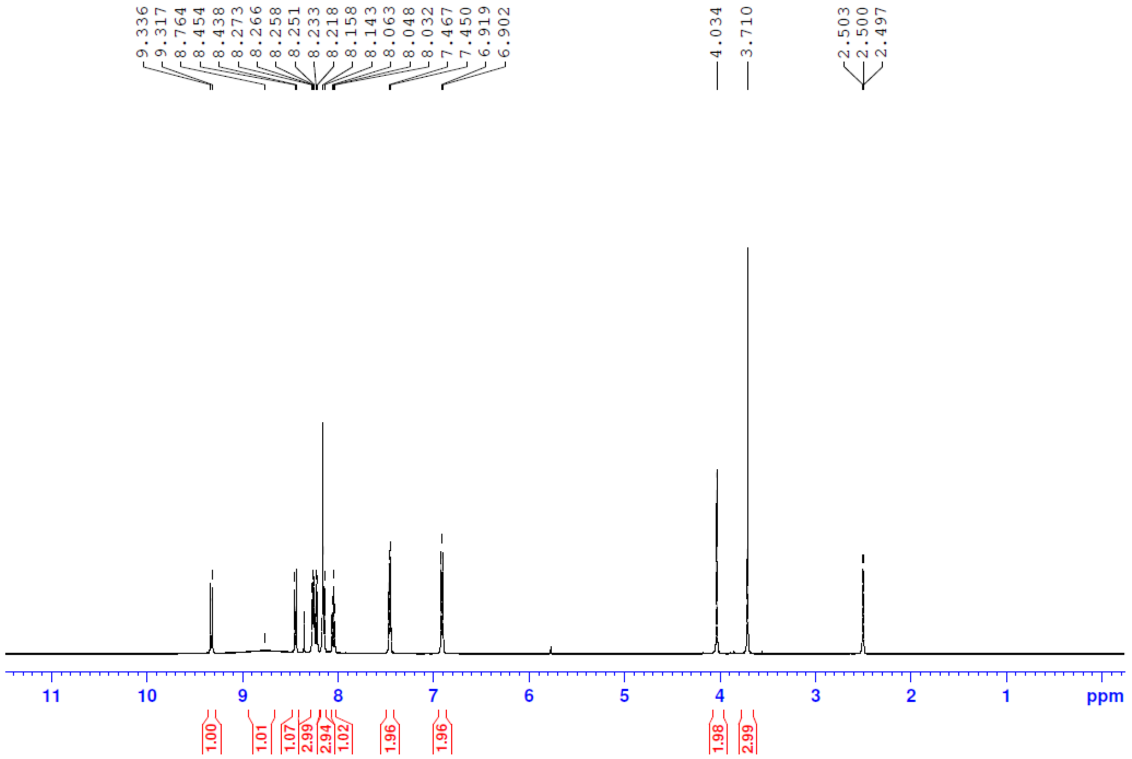

Figure S14. ^{1}H NMR spectrum of compound **8b** (500 MHz, DMSO, room temperature).

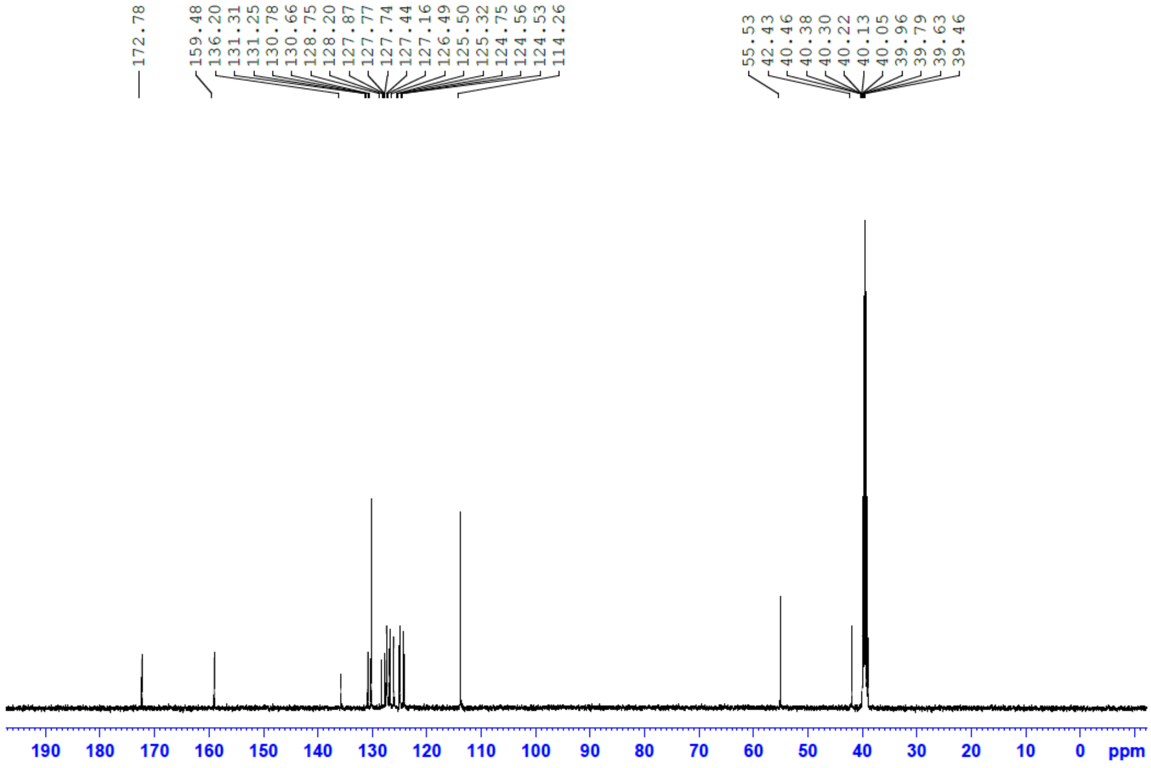

Figure S15. ^{13}C NMR spectrum of compound **8b** (125 MHz, DMSO, room temperature).

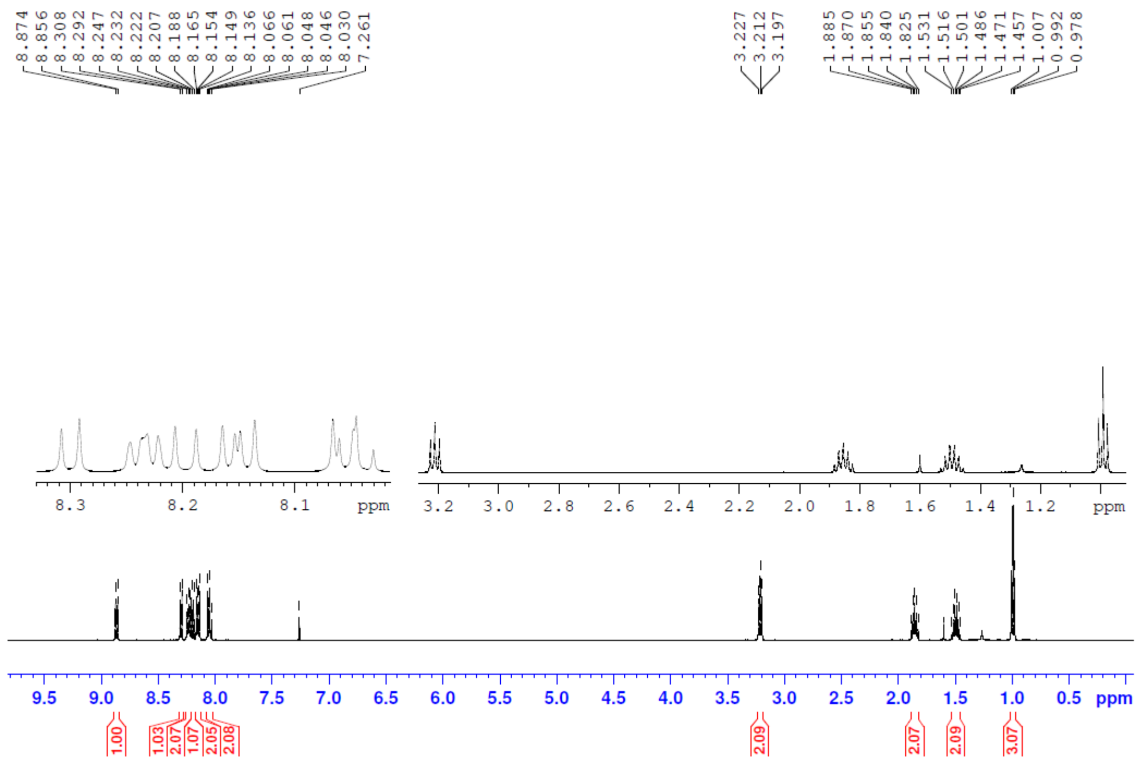

Figure S16. ^{1}H NMR spectrum of compound **9** (500 MHz, $CDCl_3$, room temperature).

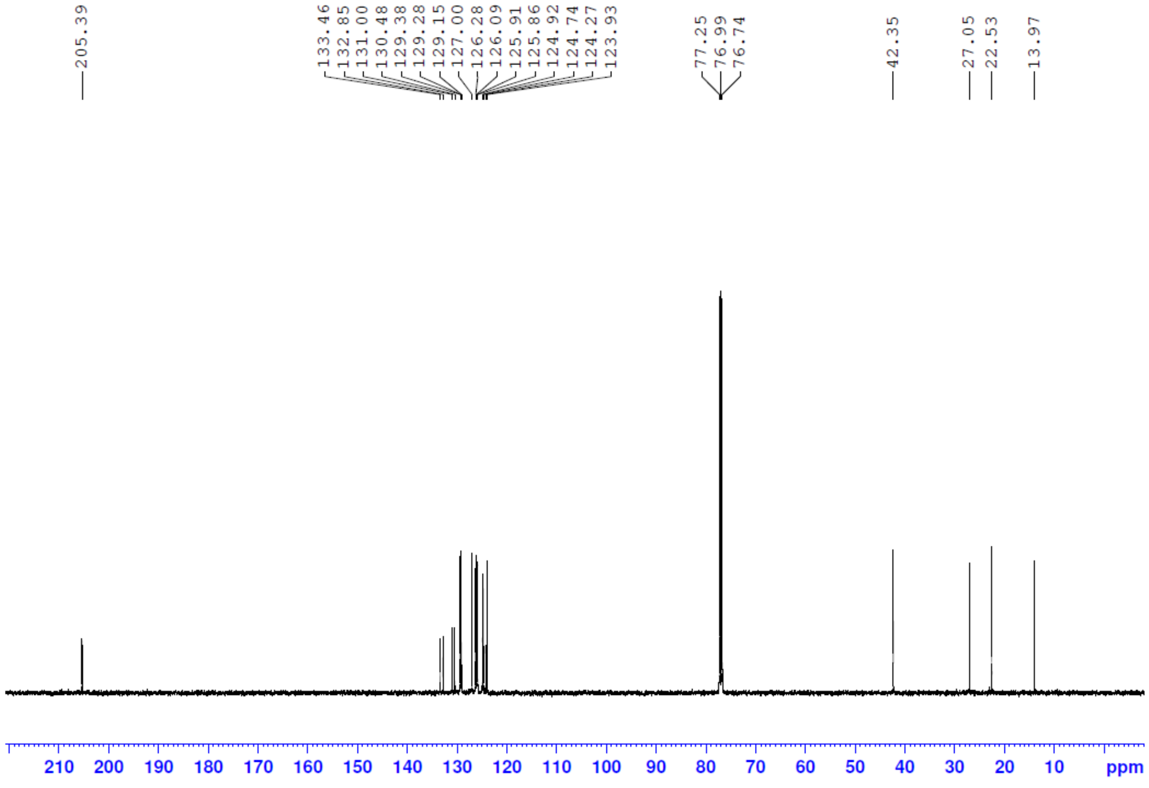

Figure S17. ^{13}C NMR spectrum of compound **9** (125 MHz, $CDCl_3$, room temperature).
